# Improved Bayesian Optimization Framework for Inverse Thermal Conductivity Based on Transient Plane Source Method

**DOI:** 10.3390/e25040575

**Published:** 2023-03-27

**Authors:** Hualin Ji, Liangliang Qi, Mingxin Lyu, Yanhua Lai, Zhen Dong

**Affiliations:** 1 School of Energy and Power Engineering, Shandong University, Jinan 250061, China; 2 Suzhou Research Institute, Shandong University, Suzhou 215123, China

**Keywords:** the transient plane source method, thermal conductivity, the Bayesian optimization algorithm, the genetic algorithm

## Abstract

In order to reduce the errors caused by the idealization of the conventional analytical model in the transient planar source (TPS) method, a finite element model that more closely represents the actual heat transfer process was constructed. The average error of the established model was controlled at below 1%, which was a significantly better result than for the analytical model, which had an average error of about 5%. Based on probabilistic optimization and heuristic optimization algorithms, an optimization model of the inverse heat transfer problem with partial thermal conductivity differential equation constraints was constructed. A Bayesian optimization algorithm with an adaptive initial population (BOAAIP) was proposed by analyzing the influencing factors of the Bayesian optimization algorithm upon inversion. The improved Bayesian optimization algorithm is not affected by the range and individuals of the initial population, and thus has better adaptability and stability. To further verify its superiority, the Bayesian optimization algorithm was compared with the genetic algorithm. The results show that the inversion accuracy of the two algorithms is around 3% when the thermal conductivity of the material is below 100 ${\mathrm{Wm}}^{-1}K^{-1}$, and the calculation speed of the improved Bayesian optimization algorithm is three to four times faster than that of the genetic algorithm.

## 1. Introduction

The thermophysical properties of a substance are observed to characterize its heat transport and heat carrying capacity, which is an important basis for material selection and thermal process analysis. The transient planar source (TPS) method is one of the most important general methods for testing the thermal conductivity and thermal diffusion coefficients, which are widely used in testing of the thermophysical properties of various materials such as, fluids [1,2,3], solids [4,5,6], powders [7,8], and thin films [9,10]. TPS provides a double helix probe (for heating and temperature sensing) and an idealized heat transfer analysis model. During the test, the thermal conductivity and thermal diffusion coefficient of the test material are obtained via iterative least squares fitting based on the transient average temperature response of the probe [11,12]. Limited by the difficulty of obtaining the analytical solution of the heat transfer model, the analytical model used for data analysis ignores the influences of probe heat capacity, thickness, thermal contact resistance, and other factors on measurement accuracy. Even with corrections made using various methods, it is difficult to achieve better breakthroughs in terms of technology, accuracy, and theory [13,14,15]. In this regard, this paper proposes combining probabilistic and heuristic optimization algorithms with the thermal conductivity differential equation as a constraint, in order to build an optimization model for the inverse problem of thermal conductivity via TPS and improve the test accuracy.

The first task in parameter inversion with a numerical technique is to establish the forward problem model in relation to the inverse process. Even though the analytical model can be used for this process, its strong idealization limits its effectiveness in characterizing thermal properties [15]. Nowadays, numerical heat transfer technology is widely used because it can meet the challenges brought about by complex problems [16,17,18]. For the TPS method, Mihiretie [19] used the finite element method to build a 3D Hot Disk model, which achieved a good match between the simulated temperature increase and the experimental results. Zhang [9] and Wang [20] et al. used the software Fluent to conduct numerical studies on the TPS used to measure thin film and translucent materials. In addition, Bording [21] and Castillo [22] et al. applied a numerical simulation to a study using a hot-wire method and obtained good test results. In order to reduce the error of the TPS model, a mathematical model that more closely represents the actual heat transfer process is established in this paper by considering the thickness and heat capacity of the probe. The model is discretized using the finite element method to solve the transient average temperature of the heater in the probe.

The forward problem model cannot directly identify parameters; thus, using the inverse heat transfer theory and the optimization algorithm, it is necessary to find the optimal parameter solution corresponding to the minimum difference between the analytical response of the forward problem and the known data. In contrast to the forward problem, the inverse problem is usually ill-posed and ill-conditioned [23,24]. Some regularization methods [25,26], such as the Levenberg–Marquardt [27] method and the conjugate gradient method [28,29], are commonly used to deal with such problems. However, it is difficult to obtain gradient information using these methods, and they easily lead to the dilemma of local optimization [30]. Different from the above deterministic methods, Bayesian reasoning technology has attracted much attention in the study of the reverse heat transfer problem due to its use of prior information and its ability to evaluate uncertainty [24]. Khan [31] and Xu [32] used Bayesian inference for the parameter estimation of steam box and textile materials and validated the effectiveness of the method. Somasundharam [33] compared three sampling techniques (Metropolis–Hastings Markov Chain Monte-Carlo, Parallel Tempering, and Evolutionary Monte-Carlo) under different noises. Helcio [24] focused on methods for solving inverse problems under Bayesian inference and the application of Markov Chain Monte-Carlo (MCMC). The core aim of Bayesian inference is to ensure that the sampling mean converges to the MCMC’s expectation of the posterior probability distribution under a large number theorem. The sampling volume of this process is huge, requiring the frequent mobilization of expensive numerical models and high computational costs. Different from Bayesian inference, the intelligent random optimization algorithm transforms parameter identification into an optimization problem that globally seeks the optimal solution of the objective function. These algorithms include the genetic algorithm [34,35], particle swarm optimization [36,37,38], social spider optimization [30], the artificial bee colony algorithm [39], Bayesian optimization [40,41], etc., all of which have achieved good results in the research on the inverse heat transfer problem. Among them, the genetic algorithm is a classic population-based heuristic algorithm that measures the goodness of fit of individuals based only on the fitness function, which can obtain a global optimal solution and is widely used in complex optimization problems [42,43]. The probability-based Bayesian optimization algorithm combines approximate metamodel technology with sampling criteria based on prior information, which enables it to carry out fast convergence. In this regard, in this paper, both methods are applied to the inverse problem model for optimizing thermal conductivity, and a comparative analysis is carried out.

The rest of the paper is arranged as follows. First, based on the principles of the TPS method and the intelligent random optimization algorithm, mathematical models of the forward and backward problems are established. Next, the correctness of the developed finite element numerical model (FENM) is verified by comparing the results with computational fluid dynamics (CFD) software using the same model. The analytical model and FENM are compared and analyzed using the calculated temperature of the CFD as the standard. The validated FENM and CFD are used simultaneously on the optimization model of the inverse thermal conductivity problem. The thermal conductivity of the solid specimen is determined using the Bayesian optimization algorithm (BOA) by employing the transient temperature response data of the probe, and the influencing factors of the inversion results are discussed. A Bayesian optimization algorithm with an adaptive initial population (BOAAIP) is proposed in the inverse architecture and compared with the genetic algorithm.

## 2. Numerical Calculation Model

### 2.1. Transient Heat Conduction of TPS

**Figure 1:**
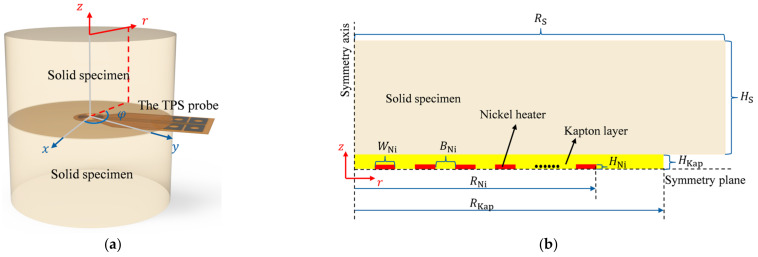
Schematic of the physical and computational domain for the TPS measurement system. (**a**) a 3D physical model demonstrates the actual measurement structure; (**b**) 2D numerical computational domain model used in this study, where the number of the rings in the nickel heater is 15 [15].

The etched double helix probe was the core element of the TPS, and it provided heat and temperature feedback during the measurements [44]. When measuring solid materials using the TPS method, the probe was sandwiched between two identical pieces of the measured material, as shown in Figure 1a. The traditional analytical model ignored the actual structure of the probe, while we considered factors such as the heat capacity of the probe in the simulation model. Because the model was strictly based on cylindrical coordinate symmetry, only 1/4 of the model was used for this research, and Figure 1b shows the simplified 2D axisymmetric heat transfer model. The relevant dimensions of the probe were constructed according to the standard Hot Disk probe [12], and Table 1 lists the relevant parameters of each component of the probe in this study.

### 2.2. Governing Equations

Based on the above 2D axisymmetric model, the transient thermal conductivity process of TPS is calculated numerically. When the internal heat source is considered, the governing equation of heat conduction in a cylindrical coordinate system is shown in Equation (1) [45]:

(1) $$\rho c_{v}\frac{\partial T}{\partial t}=\frac{1}{r}\frac{\partial }{\partial r}\left(\lambda r\frac{\partial T}{\partial r}\right)+\frac{\partial }{\partial z}\left(\lambda \frac{\partial T}{\partial z}\right)+\Theta \;$$

where λ is the thermal conductivity ${\mathrm{Wm}}^{-1}K^{-1}$; ρ is the density ${\mathrm{kgm}}^{-3}$; $c_{v}$ is the volume-specific heat capacity ${\mathrm{Jkg}}^{-1}K^{-1}$; $\frac{\partial }{\partial r}\left(\lambda r\frac{\partial T}{\partial r}\right)$ and $\frac{\partial }{\partial z}\left(\lambda \frac{\partial T}{\partial z}\right)$ are the temperature gradients in the r and z directions, respectively; and Θ is the source item.

**Figure 2:**
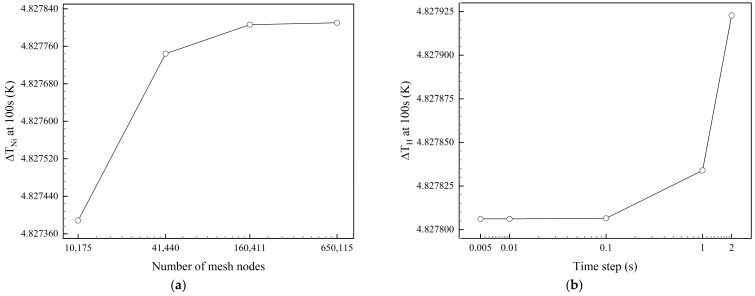
Numerical accuracy verification. (**a**) Mesh independence verification; (**b**) time step independence verification.

The finite element method is used to discretize Equation (1) to obtain the backward difference format as shown in Equation (2) [46]:

(2) $$\left(M_{\lambda }+\frac{1}{\Delta t}M_{C}\right)T\left(t_{n+1}\right)=Q\left(t_{n+1}\right)+\frac{1}{\Delta t}M_{C}T\left(t_{n}\right)\;$$

where $M_{\lambda }$ is the thermal conductivity matrix of the calculation units; $M_{C}$ is the heat capacity matrix of the calculation units; Δt is the time step s; $T\left(t_{n+1}\right)$ and $T\left(t_{n}\right)$ are the node temperatures at moments $t_{n+1}$ and $t_{n}$, respectively, K; and $Q\left(t_{n+1}\right)$ is the heat load generated by the internal heat in the unit at time $t_{n+1}$ W. In order to consider the influence of mesh and time on accuracy, the irrelevance of both is verified using a ceramic material ($\lambda =1.50\;{\mathrm{Wm}}^{-1}K^{-1},\;\kappa =0.59\;{\mathrm{mm}}^{2}s^{-1}$). Figure 2 shows that the calculated temperature increase in the heat source at 100 s tends to stabilize and no longer fluctuates significantly when the number of nodes for the mesh reaches 160,411 and the time step is less than 0.1 s. Considering the accuracy and cost of the calculation, the mesh node and time step are set at 160,411 and 0.01 s, respectively.

### 2.3. Boundary and Setting Conditions

The model’s boundary settings and conditions were as follows. The left side and bottom of the whole model: the symmetry axis and symmetry plane for the bottom surface, respectively. The outer boundaries of the solid specimen and the right side of the Kapton layer: thermal isolation. In the transient heat transfer process of TPS, the solid specimen is large enough that the heat will not penetrate the sample material during the heating time, thus this part of the boundary can be used for thermal isolation; a study by Zheng et al. [15] found that convection and radiation have little effect on the test system. In the simulation process, the power of the heater was given, and the initial ambient temperature was 293.15 K.

## 3. Thermal Conductivity Identification Based on an Optimization Algorithm

**Figure 3:**
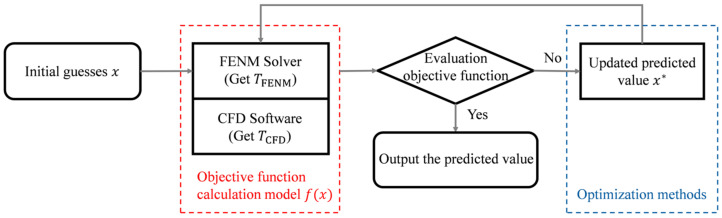
Flow chart of thermal conductivity identification by optimization algorithm.

The process of identifying thermal conductivity using the optimization algorithm is shown in Figure 3. In the identification process, the real thermal conductivity of the sample is input into CFD software, and the obtained transient temperature responses $T_{\mathrm{CFD}}$ are taken as the real data. The developed finite element numerical model (FENM) is used as a temperature solver, and its simulated temperature $T_{\mathrm{FENM}}$ is used as the prediction data (consider all simulation data to be obtained within 0–10 s). The objective function is established according to the mean square error of the real data $T_{\mathrm{CFD}}$ and the predicted data $T_{\mathrm{FENM}}$:

(3) $$f\left(x\right)=\frac{1}{N}\overset{N}{\underset{i=1}{\sum }}{\left(T_{\mathrm{FENM},i}\left(x\right)-T_{\mathrm{CFD},i}\right)}^{2}$$

where x represents the parameter variable, i.e., the thermal conductivity of the specimen; i denotes a measuring point; and N is the total number of measuring points. The parameter identification process is transformed into an optimization problem that seeks the global optimal solution of the objective function [41]:

(4) $$x^{*}={\mathrm{argmin}}_{x\in \chi \subseteq {\mathbb{R}}^{d}}f\left(x\right)$$

where x has the same meaning as above; χ is the observation space; $f\left(x\right)$ is the above objective function; f: ${\mathbb{R}}^{d}\to \mathbb{R}$; and $x^{*}$ is the current optimal estimate.

Although the objective function can be defined, the corresponding objective function value can only be calculated according to the discrete independent variable. The optimization of the objective function is known as a “black box” optimization problem, from which it is difficult to obtain effective gradient information, and the evaluation of objective function is expensive. In this paper, the optimization models for the inverse heat transfer problem are investigated using Bayesian optimization and the genetic algorithm based on the gradient-free stochastic optimization theory.

### 3.1. Bayesian Optimization Algorithm

The Bayesian optimization framework is used to establish and update the probabilistic surrogate model based on previous evaluations of the objective function [47], and to actively select the evaluation points with the most global “potential” through the acquisition function. Bayesian optimization can effectively use prior information to judge the uncertainty of the unknown region and obtain the optimal solution within a few evaluations. This study develops a probabilistic agent model for the objective function $f\left(x\right)$ based on the Gaussian process.

The Gaussian process is a paradigm of a multivariate Gaussian probability distribution and is mainly composed of the mean function m and covariance function k [48]:

(5) $$f\left(x\right)\sim GP\left(m\left(x\right),k\left(x,x^{\prime }\right)\right)$$

(6) $$m\left(x\right)=E\left[f\left(x\right)\right]$$

(7) $$k\left(x,x^{\prime }\right)=E\left[\left(f\left(x\right)-m\left(x\right)\right)\left(f\left(x^{\prime }\right)-m\left(x^{\prime }\right)\right)\right]$$

where x is the thermal conductivity. When there is observation noise, the observed values (objective function values with noise) are $y=f\left(x\right)+\varepsilon$, and we can suppose that the noise ε satisfies $p\left(\varepsilon \right)=N\left(0,\sigma^{2}\right)$ [49]. In this regard, according to the definition of the Gaussian process, the joint distribution of Gaussian variables can be obtained as follows [50]:

(8) $$\left[\begin{array}{c} Y\\ f\left(x^{*}\right) \end{array}\right]\sim N\left(\left[\begin{array}{c} \begin{array}{c} m\left(x_{1}\right)\\ \vdots \end{array}\\ \begin{array}{c} m\left(x_{t}\right)\\ m\left(x^{*}\right) \end{array} \end{array}\right],\left[\begin{array}{cc} K+\sigma^{2}I & k\left(x^{*},X\right)\\ k{\left(x^{*},X\right)}^{T} & k\left(x^{*},x^{*}\right) \end{array}\right]\right)\;$$

where X is the training input set $x_{1:t}$, Y is the training output set $y_{1:t}$, K is the matrix of covariance functions $k\left(x,x^{\prime }\right)$, and I is the unit matrix. To obtain the posterior predictive distribution of $f\left(x^{*}\right)$, the test point $x^{*}$ and training dataset D are set as follows [51]:

(9) $$p\left(f\left(x^{*}\right)|x^{*},D\right)=\frac{p\left(f\left(x^{*}\right),Y|X,x^{*}\right)}{p\left(Y|X\right)}$$

The mean and variance define the conditional posterior Gaussian distribution, according to Equation (8), the following distribution can be obtained [52]:

(10) $$\mu \left(f\left(x^{*}\right)|x^{*},D\right)=m\left(x^{*}\right)+k{\left(x^{*},X\right)}^{T}{\left(K+\sigma^{2}I\right)}^{-1}\left(Y-\left[m\left(X_{:,1}\right)\right],\ldots ,m\left(X_{:,nD}\right)\right)$$

(11) $$var\left(f\left(x^{*}\right)|x^{*},D\right)=k\left(x^{*},x^{*}\right)-k{\left(x^{*},X\right)}^{T}{\left(K+\sigma^{2}I\right)}^{-1}k\left(x^{*},X\right)$$

The $k\left(x,x^{\prime }\right)$ is defined using various kernel functions. The squared exponential kernel function chosen in this study is infinitely differentiable, can be derived infinitely, is always continuous, and has two hyperparameters $\theta_{1}$ and $\theta_{2}$ [51]:

(12) $$k\left(x-x^{\prime }\right)=\theta_{1}{}^{2}exp\left(-\frac{∥x-x^{\prime }{∥}^{2}}{2\theta_{2}{}^{2}}\right)$$

There are many types of acquisition function, and, in this study, the expected improvement is used [53]:

(13) $$EI\left(x\right)=\left\{\begin{array}{c} \left(f\left(x^{*}\right)-\mu_{t}\left(x\right)-\xi \right)\Phi \left(Z\right)+\sigma_{t}\left(x\right)\phi \left(Z\right),\sigma_{t}\left(x\right)>0\\ \;0\;\;\;\;\;\;\;\;\;\;\;\;\;\;\;\;\;\;\;\;\;\;\;\;\;\;\;\;\;\;\;\;\;\;\;\;\;\;\;\;\;\;\;\;\;\;\;\;\;\;\;\;\;\;\;\;\;\;\;\;\;\;\;\;\;\;\;\;\;\;\;\;,\;\sigma_{t}\left(x\right)=0\; \end{array}\right.$$

(14) $$Z=\frac{f\left(x^{*}\right)-\mu_{t}\left(x\right)-\xi }{\sigma_{t}\left(x\right)}$$

where $f\left(x^{*}\right)$ is the objective function value of the current evaluation point; $\Phi \left(\cdot \right)$ and $\phi \left(\cdot \right)$ are the standard Gaussian probability density and cumulative density functions, respectively; $\mu_{t}\left(x\right)$ and $\sigma_{t}\left(x\right)$ are the expectation and variance of the Gaussian distribution at x, respectively; ξ is the equilibrium parameter (used to balance the relationship between the local and global search).

### 3.2. Optimization Validation

The developed Bayesian optimization algorithm (BOA) was verified via the six-hump camel back problem on the MATLAB website. The six-hump camel back function has multiple extremums in the region [−3,3], and its expression is shown below [54]:

(15) $$sinmin=4x_{1}{}^{2}-2.1x_{1}{}^{4}+\frac{x_{1}{}^{6}}{3}+x_{1}x_{2}-4x_{2}{}^{2}+4x_{2}{}^{4}$$

**Figure 4:**
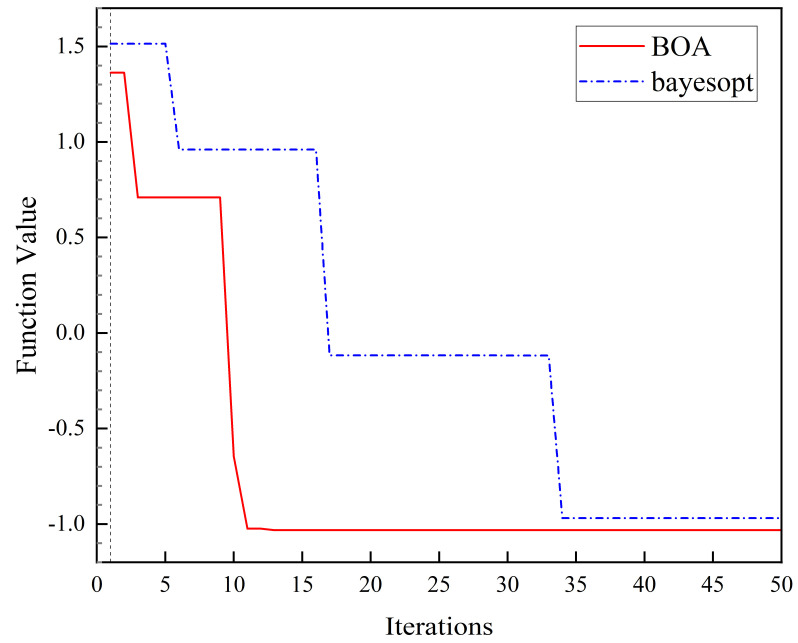
Minimum function values and iterations.

The developed BOA, together with the Bayesian optimizer bayesopt and the global optimizer GlobalSearch in MATLAB (MathWorks, Natick, MA, USA), will jointly search for the global minimum solution of this function in the region [−3,3] simultaneously. In the study, all of the GlobalSearch settings were kept consistent with the official case, and its optimization results were used to check the correctness of the BOA and bayesopt settings [55]. The initial population of both BOA and bayesopt was 30, and the maximum number of iterations was 50. The difference is that the acquisition function of BOA was “expected-improvement”, while that of bayesopt was “expected-improvement-plus” [56]. Table 2 shows the optimization results of the three methods used for this case. Figure 4 shows the comparison curves of the observed minimum objective function values for the BOA and bayesopt within 30 iterations. It can be seen from Table 2 that the optimization results of the developed BOA were the same as those of GlobalSearch; the minimum function value optimized via bayesopt was close to that of GlobalSearch, but there was a certain gap between the optimized variable $x_{1}$ and the real solution. As can be seen in Figure 4, the minimum objective function value in the initial sample of bayesopt is higher than that of BOA, and the overall minimum objective function values of both are observed to approach the true minimum. The comprehensive comparison of BOA with bayesopt and GlobalSearch was sufficient to verify the correctness of the developed BOA.

### 3.3. A Bayesian Optimization Algorithm with an Adaptive Initial Population

Every evaluation of the target function requires the mobilization of the expensive FENM, which can take a lot of time when the initial range of parameters is too large. In the inversion process, reducing the initial range of parameters can effectively improve operational efficiency. The temperature response of the TPS probe is affected by a variety of thermophysical properties of solid specimens. In this study, only a single-parameter inversion of thermal conductivity was performed. Under this model, the transient average temperature of the probe decreases with the increasing thermal conductivity of the specimen when the experimental conditions are the same [57]. Therefore, the range evaluation function is defined as follows:

(16) $$g\left(x\right)=\frac{1}{N}\overset{N}{\underset{i=1}{\sum }}\left(T_{\mathrm{FENM},i}\left(x\right)-T_{\mathrm{CFD},i}\right)$$

**Figure 5:**
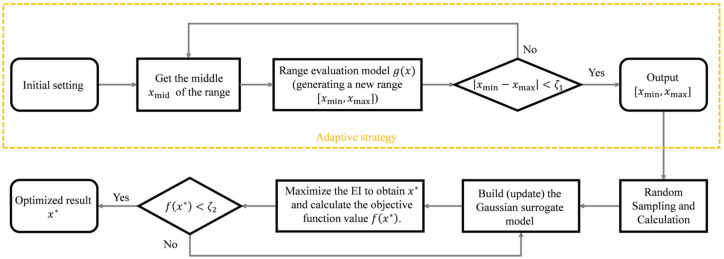
Flowchart of the BOAAIP algorithm.

When the initial range $\left[x_{\min},x_{\max}\right]$ is given, the range evaluation function satisfies $g\left(x_{\min}\right)g\left(x_{\max}\right)<0$. For this property, a Bayesian optimization algorithm with an adaptive initial population (BOAAIP) is proposed based on the dichotomous method. Figure 5 shows the complete framework of the algorithm, in which the difference between BOAAIP and the traditional BOA lies in the “dichotomous strategy”, as shown in the yellow box.

The specific process is as follows:

(1) Input the initial range, the accuracy $\zeta_{1}$ of the range evaluation function, the accuracy $\zeta_{2}$ of the objective function, and other initial conditions.
(2) Calculate the middle value $x_{\mathrm{mid}}$ of the given range and input the range evaluation model $g\left(x\right)$ to update the range. The updated logic is as follows: if $g\left(x_{\mathrm{mid}}\right)<0$ then $x_{\max}=x_{\mathrm{mid}}$; otherwise, $x_{\min}=x_{\mathrm{mid}}$.
(3) Determine whether $\left|x_{\min}-x_{\max}\right|$ satisfies the precision $\zeta_{1}\;\left(\zeta_{1}=10\right)$, and, if so, output the new range; otherwise, return to step (2).
(4) Perform random sampling in the new range and bring the samples into the forward problem model to obtain the training input set X and the training output set Y.
(5) Build and update the agent model based on the Gaussian process.
(6) Maximize the EI acquisition function, obtain the next prediction point $x^{*}$, and calculate the objective function value $f\left(x^{*}\right)$.
(7) Determine whether the value of the objective function satisfies precision $\zeta_{2}\;\left(\zeta_{2}=0.001\right)$; if so, output the optimization result. Otherwise, return to step (5).

### 3.4. Genetic Algorithm

In order to study the performance of probability and heuristic algorithms in the optimization of inverse thermal conductivity problems, the genetic algorithm is used as a scheme for comparison with Bayesian optimization. The genetic algorithm is a classic population-based heuristic algorithm that starts with any initial population and evolves it to improve the quality of the results through continuous evolution [42]. In this study, secondary development was carried out based on the source code provided by [58]; in this case, 20 to 100 individuals were randomly selected within the range given by the dichotomy strategy, and the individuals in the population were binary-coded to form the initial population. To guarantee the optimization direction, we selected the reciprocal of the objective function as the fitness function:

(17) $$fit\left(x\right)=\frac{1}{\frac{1}{N}\sum_{i=1}^{N}{\left(T_{\mathrm{FENM},i}\left(x\right)-T_{\mathrm{CFD},i}\right)}^{2}}$$

The roulette selection method is used as the selection operator, and the single-point cross method is used to update the chromosomes. With binary coding, the variation ranges from 0 to 1 or 1 to 0. In the inverse problem optimization model, global optimization is achieved by continuously evaluating the fitness function value of the individual populations.

## 4. Analysis and Discussion

### 4.1. Correctness Verification and Accuracy Comparison

**Figure 6:**
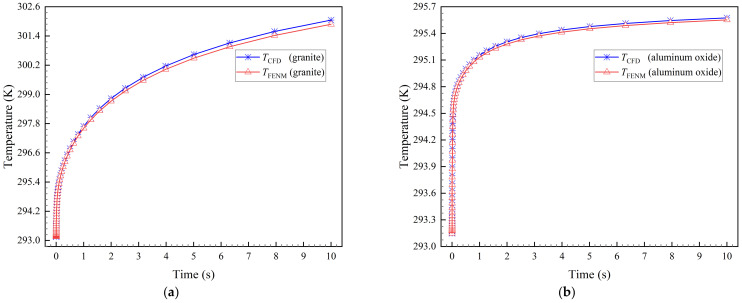

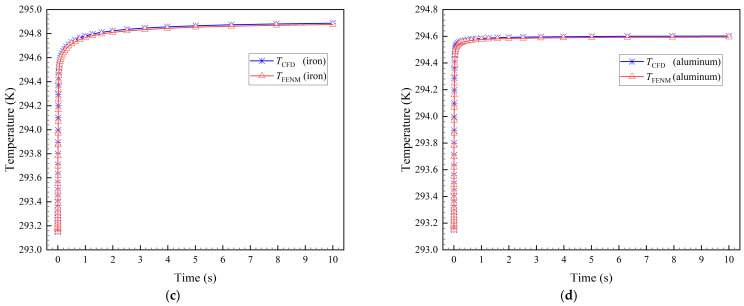
Comparison of simulation results of solid specimens. (**a**) Comparison of simulation results for granite; (**b**) comparison of simulation results for aluminum oxide; (**c**) comparison of simulation results for iron; (**d**) comparison of simulation results for aluminum.

For the forward problem, the unstructured meshing of the 2D model was performed using MATLAB based on DistMesh [59] (a simple mesh generator), and a numerical study was carried out using the finite element method. Before carrying out the inversion study, it was necessary to verify the correctness of the FENM. The four materials in Table 3 were used as test specimens, and the simulation results obtained using the FENM and CFD software were compared under the same settings. Figure 6 shows a comparison of the simulation results obtained using each method. It can be seen that the difference between the two calculation results is small, with a maximum relative error below 2%, which verifies the reliability of the FENM.

**Figure 7:**
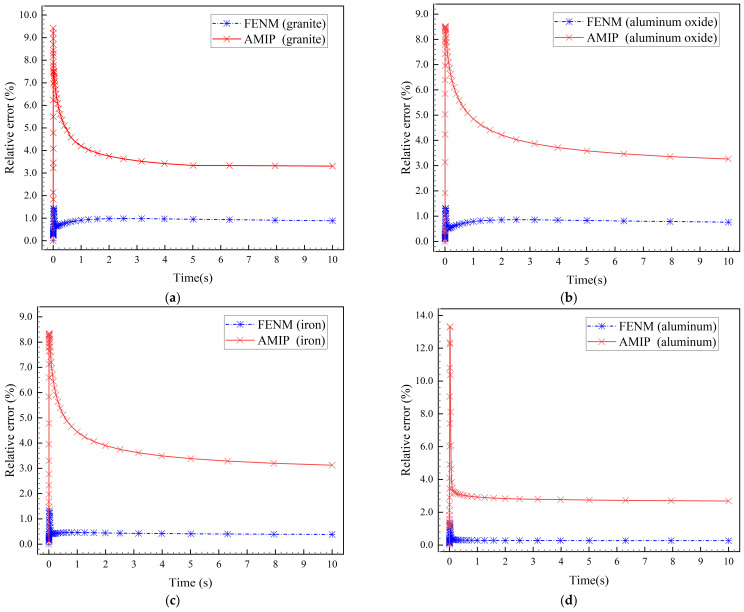
Comparison of relative errors of FENM and AMIP. (**a**) Comparison results of granite; (**b**) comparison results of aluminum oxide; (**c**) comparison results of iron; (**d**) comparison results of aluminum.

The analytical model for solving thermal conductivity using the TPS method is shown in Equation (18) [60]:

(18) $$\Delta T_{\mathrm{Ni}}\left(\tau \right)=\frac{P_{0}}{\pi^{3/2}R_{\mathrm{Ni}}\lambda_{S}}H\left(\tau \right)$$

where τ is the dimensionless time; $H\left(\tau \right)$ is the dimensionless time function; $P_{0}$ is the heating power of the probe W; $\lambda_{S}$ is the thermal conductivity of the specimen ${\mathrm{Wm}}^{-1}K^{-1}$; $R_{\mathrm{Ni}}$ is the radius of the probe m; and $\Delta T_{\mathrm{Ni}}\left(\tau \right)$ is the average temperature increase in the probe K. In order to compare the analytical accuracy of the model, the temperature data of the CFD software were brought into the self-developed analytical model identification program (AMIP). The analytical solution of the response temperature was calculated by fitting the obtained H(τ) and the slope parameter. Using the temperature data of CFD as the standard, the calculation relative errors of FENM and AMIP were compared and analyzed, and the comparison results are shown in Figure 7. As can be seen in Figure 7, regarding the four different specimens, the maximum relative errors of AMIP and CFD are 9.42%, 8.51%, 8.34%, and 13.31%, respectively; the average relative errors are 5.72%, 5.69%, 5.62%, and 4.11%, respectively. Regarding these four different specimens, the maximum relative errors of FENM and CFD are 1.43%, 1.31%, 1.32%, and 1.33%, respectively, and the average relative errors are 0.71%, 0.59%, 0.47%, and 0.41%, respectively. It can be seen that the average calculation error of FENM is below 1%, which is a much better result than that of the traditional analysis model (5%).

### 4.2. Bayesian Optimization Results

A hypothetical solid specimen HS ($\lambda_{\mathrm{HS}}=5.00\;{\mathrm{Wm}}^{-1}K^{-1}$, $\kappa_{\mathrm{HS}}=1.15\;{\mathrm{mm}}^{2}s^{-1}$) was chosen to analyze the factors affecting the inversion results of the BOA algorithm. The methods and conditions described in the positive problem model above were used for the simulation, and the corresponding prediction data $T_{\mathrm{FENM}}$ and real data $T_{\mathrm{CFD}}$ were obtained. In this study, three initial population ranges ($R_{\mathrm{ip}1}=1-10\;{\mathrm{Wm}}^{-1}K^{-1}$, $R_{\mathrm{ip}2}=1-30\;{\mathrm{Wm}}^{-1}K^{-1}$, and $R_{\mathrm{ip}3}=1-50\;{\mathrm{Wm}}^{-1}K^{-1}$) were selected, and three groups of population individuals (5, 10, and 15) were set.

**Figure 8:**
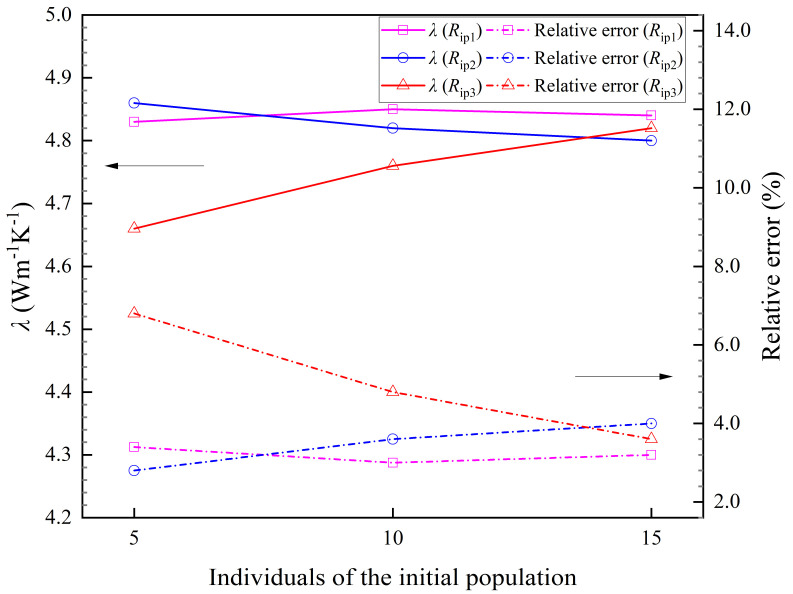
Effect of different initial population ranges and individuals on inversion results.

Figure 8 shows the effect of different initial population ranges and individuals on the inversion results. It can be seen that the relative error tends to decrease as the number of individuals in the population increases when the initial population range is $R_{\mathrm{ip}3}$; when the initial population range is $R_{\mathrm{ip}1}$ and $R_{\mathrm{ip}2}$, the change in population individuals does not significantly affect the relative error; moreover, there is no evident effect of variation in the initial population range on relative error with the same population individuals.

**Figure 9:**
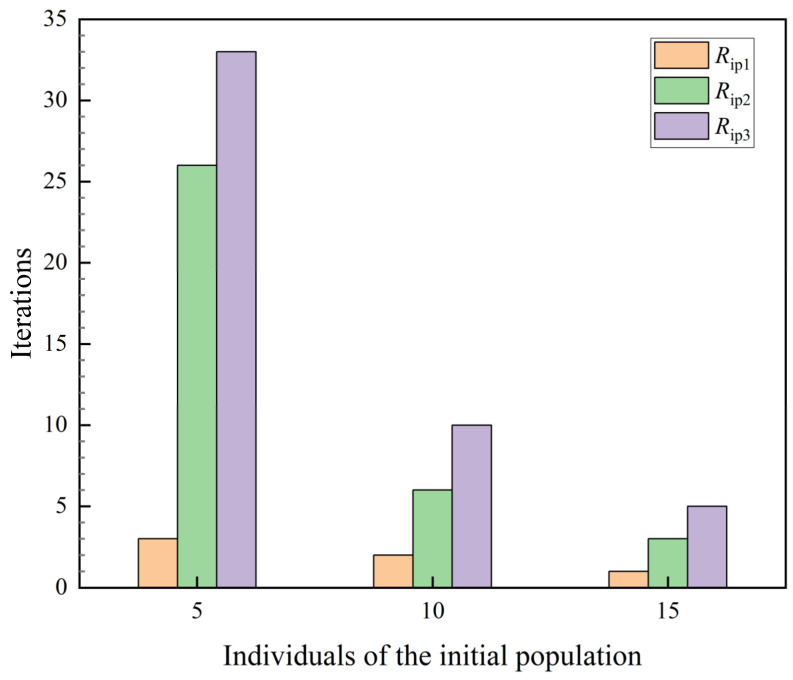
Effect of different initial population ranges and individuals on the number of iterations.

Figure 9 shows the effect of different initial population ranges with individuals on the number of iterations. It can be seen that the number of iterations increases as the initial population range increases when the initial population has the same individuals; moreover, for the same initial population range, the number of iterations decreases as the number of individuals in the initial population increases.

### 4.3. Optimization Results after Algorithm Improvement

In order to study the optimization results after the algorithm’s improvement, simulations were performed in the BOAAIP framework with the solid specimen HS assumed above (the initial population of individuals was set to 5).

**Figure 10:**
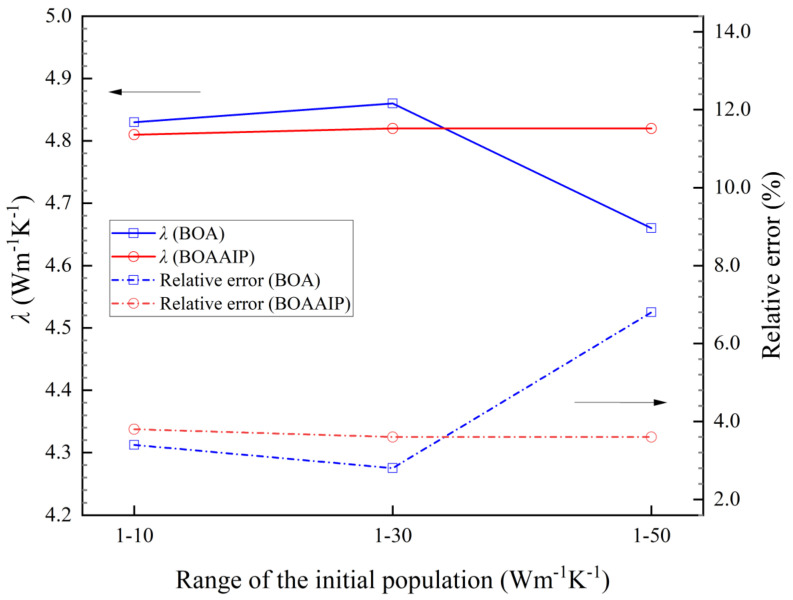
Comparison of inversion results under different initial population ranges.

**Figure 11:**
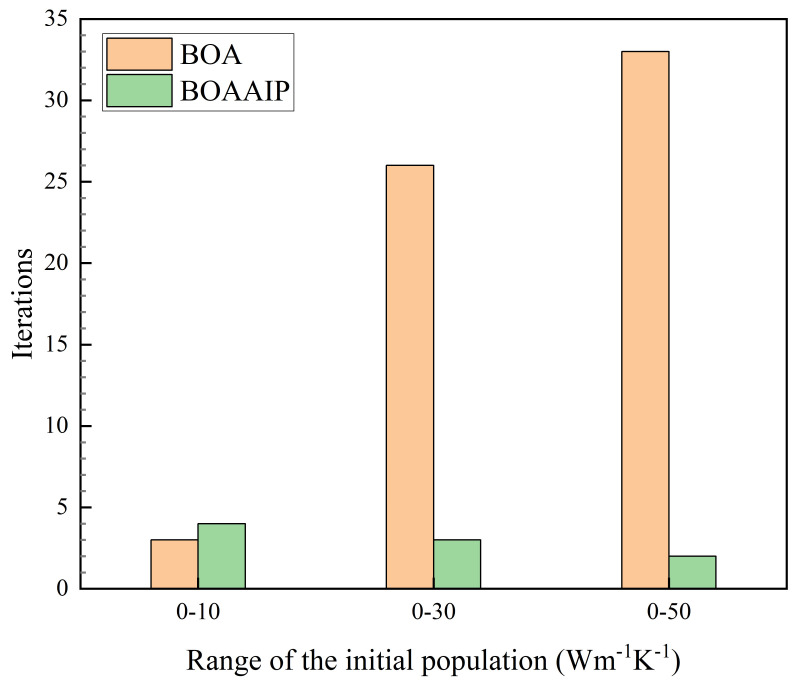
Comparison of the number of iterations under different initial population ranges.

The data containing five individuals in BO were selected for analysis and comparison with the research results of BOAAIP. Figure 10 compares the inversion results of the two algorithms at different initial population ranges. The inversion results of BOA showed irregular fluctuations, and there were evident differences in the inversion results under different initial population ranges; moreover, the inversion results of BOAAIP were sta–ble, and the average error was controlled at below 4%. Figure 11 shows a comparison of the number of iterations of the two algorithms at different initial population ranges. It can be seen that there is a clear increasing trend in the number of iterations of BOA with an increase in the initial population range. However, the number of iterations of BOAAIP decreases slightly with an increase in the initial population range, the overall performance is stable, and it converges within five iterations.

### 4.4. Algorithm Comparison

In order to further verify the feasibility of the proposed optimization framework, BOAAIP and GA were used to compare the inversion results of the solid specimens, and the results are shown in Table 4. The population range in the GA was determined in the same way as in the BOAAIP. The number of individuals in the population was set to 30, binary coding was used, and the encoding length was set to 10. The selection method was a roulette selection method with a crossover probability of 0.5, a variance probability of 0.05, and a maximum number of iterations of 1000.

**Figure 12:**
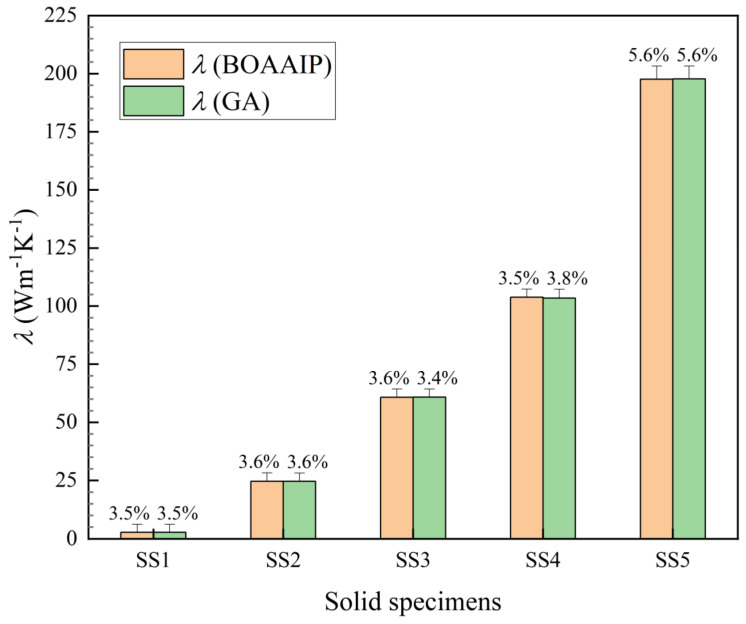
Comparison of the inversion results of five solid specimens using the two algorithms.

Figure 12 compares the inversion results of five solid specimens using both BOAAIP and GA. According to the comparison of relative errors, the inversion accuracies of these two algorithms are relatively close, with no significant differences. The thermal conductivity levels of the five solid specimens selected for the study show an increasing trend, and the relative errors of both algorithms are around 3% when the thermal conductivity of the specimens is below 100 ${\mathrm{Wm}}^{-1}K^{-1}$; when the thermal conductivity of the specimen exceeds 100 ${\mathrm{Wm}}^{-1}K^{-1}$, the relative errors of both algorithms are close to 6%. This shows that the two algorithms have the same accuracy, and the proposed algorithm model is more suitable for materials with thermal conductivity below 100 ${\mathrm{Wm}}^{-1}K^{-1}$.

**Figure 13:**
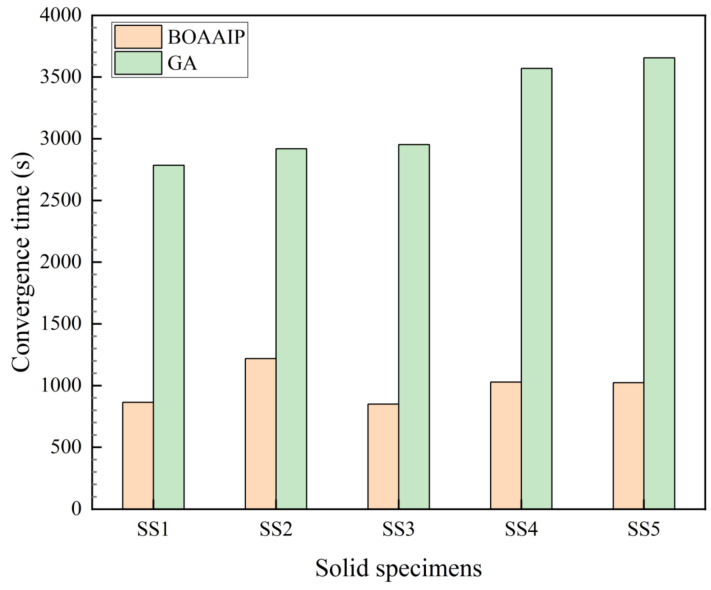
Comparison of inversion convergence time of five solid specimens using the two algorithms. Note: the operating environments of the two inversion algorithms are the same, with the CPU model being a 3.79 GHz AMD Ryzen 5 3600X 6-Core Processor, with MATLAB accounting for 9.5% of the CPU memory during the running process.

Figure 13 compares the convergence time of the inversion of five solid specimens for both BOAAIP and GA. According to the time comparison of each specimen, the convergence time of BOAAIP is significantly shorter than that of GA, and its operating speed is about three to four times faster than that of GA. Additionally, the magnitude of the thermal conductivity of the specimen has no significant effect on the calculation speed of either algorithm.

## 5. Conclusions

In order to improve the measurement accuracy of the TPS method, a complete inversion framework was developed to identify the thermal conductivity of solid specimens by combining numerical calculations and optimization algorithms. The main conclusions are as follows:

(1) The finite element numerical model of TPS, established by comprehensively considering the thickness and heat capacity of the probe, had high computational accuracy. The test results for the four materials showed that the average relative error of FENM was below 1%, and its accuracy was much higher than that of the analytical model, which had an average error of over 5%.
(2) The number of iterations of the Bayesian optimization algorithm (BOA) was susceptible to changes in the range and individuals of the initial population. However, the Bayesian optimization algorithm with an adaptive initial population (BOAAIP) was not affected by the initial population range and individuals, and its calculation results were more stable. The test results of HS (a hypothetical material) showed that the average error of BOAAIP was below 4% and that the algorithm can reach convergence within five iterations, possessing a faster computational speed compared to BOA.
(3) The computational speed of BOAAIP was much faster than that of the genetic algorithm (GA), and both models had the same accuracy. When the thermal conductivity of the solid specimens was below 100 ${\mathrm{Wm}}^{-1}K^{-1}$, the relative error of both algorithms was about 3%, but the calculation speed of BOAAIP was three to four times faster than that of GA.

## Figures and Tables

**Table 1:** Calculation parameters of the TPS probe.

Parameter	Unit	Value
$W_{\mathrm{Ni}\;}/\;B_{\mathrm{Ni}}\;/\;H_{\mathrm{Ni}}\;/\;R_{\mathrm{Ni}\;}/\;H_{\mathrm{Kap}}\;/\;R_{\mathrm{Kap}}$	mm	0.21/0.21/0.01/6.40/0.02/10
$C_{\mathrm{Ni}}/\;C_{\mathrm{Kap}}$	${\mathrm{MJm}}^{-3}K^{-1}$	4.10/1.56
$\lambda_{\mathrm{Ni}\;}/\;\lambda_{\mathrm{Kap}}$	${\mathrm{Wm}}^{-1}K^{-1}$	91.74/0.50
$\kappa_{\mathrm{Ni}}\;/\;\kappa_{\mathrm{Kap}}$	${\mathrm{mm}}^{2}s^{-1}$	22.30/0.32

**Table 2:** Optimization results of different methods.

Methods	$\mathrm{Optimal}\;\mathrm{Variable}\;x_{1}$	$\mathrm{Optimal}\;\mathrm{Variable}\;x_{2}$	Minimum Function Value
BOA	−0.089	0.712	−1.031
bayesopt	−0.016	0.776	−0.968
GlobalSearch	−0.089	0.712	−1.031

**Table 3:** Calculation parameters of solid specimens.

Parameter	Unit	Sample (Granite/Aluminum Oxide/Iron/Aluminum)
$R_{S}$	mm	70
$H_{S}$	mm	70
$C_{S}$	${\mathrm{MJm}}^{-3}K^{-1}$	2.21/3.51/3.46/2.43
$\lambda_{S}$	${\mathrm{Wm}}^{-1}K^{-1}$	2.90/27.00/76.20/238.00
$\kappa_{S}$	${\mathrm{mm}}^{2}s^{-1}$	1.30/7.69/22.00/97.90

**Table 4:** Calculation parameters of solid specimens.

Parameter	Unit	Sample (SS1/SS2/SS3/SS4/SS5)
$R_{S}$	mm	70
$H_{S}$	mm	70
$C_{S}$	${\mathrm{MJm}}^{-3}K^{-1}$	2.21/3.50/3.64/3.23/3.43
$\lambda_{S}$	${\mathrm{Wm}}^{-1}K^{-1}$	2.90/25.60/63.04/107.60/209.40
$\kappa_{S}$	${\mathrm{mm}}^{2}s^{-1}$	1.31/7.31/17.30/33.30/61.10

Note: among the solid specimens listed in Table 4, the range of the initial population for SS1 (granite), SS2 (common cupronickel B30), and SS3 (carbon steel 10) was 0-100 ${\mathrm{Wm}}^{-1}K^{-1}$; for SS4 (common brass H62) and SS5 (common cupronickel B1), the range was 0-500 ${\mathrm{Wm}}^{-1}K^{-1}$.

## Data Availability

Not applicable.

## Nomenclature

$B_{\mathrm{Ni}}$	Spacing of the nickel heater: mm
$c_{v}$	Volumetric specific heat capacity, ${\mathrm{Jkg}}^{-1}K^{-1}$
C	Volume-specific heat capacity, ${\mathrm{Jm}}^{-3}K^{-1}$
d	Dimensions of variables
D	Training dataset
$f\left(\cdot \right)$	Objective function
$fit\left(\cdot \right)$	Fitness function
$g\left(\cdot \right)$	Range evaluation function
H	Thickness of a layer, mm
$H\left(\tau \right)$	Dimensionless time function
i	A measuring point
I	Unit matrix
k	Covariance function
K	Matrix of covariance functions $k\left(x,x^{\prime }\right)$
m	Mean function
$M_{C}$	Heat capacity matrix
$M_{\lambda }$	Thermal conductivity matrix
N	The total number of measuring points
p	Probability distribution
$P_{0}$	Heating power of the probe, W
Q	Heat load generated, W
$\left(r,\varphi ,z\right)$	Cylindrical coordinates
$R_{\mathrm{ip}1}$, $R_{\mathrm{ip}2}$, $R_{\mathrm{ip}3}$	Three initial population ranges, ${\mathrm{Wm}}^{-1}K^{-1}$
$R_{\mathrm{Kap}}$	Outer radius of the Kapton layer, mm
$R_{\mathrm{Ni}\;}$	Outer radius of the last nickel heater, mm
$R_{S\;}$	Outer radius of the specimen, mm
ℝ	Real number field
t	Moment, s
Δt	Time step, s
$\Delta T_{\mathrm{Ni}}$	Transient average temperature increase in the probe, K
T	Temperature, K
$T_{\mathrm{CFD}}$	Transient average temperature obtained using the CFD software, K
$T_{\mathrm{FENM}}$	Transient average temperature obtained using the FENM, K
$var\left(\cdot \right)$	Covariance function
$W_{\mathrm{Ni}\;}$	Width of the nickel heater, mm
x	The parameter variable, i.e., the thermal conductivity of the specimen, ${\mathrm{Wm}}^{-1}K^{-1}$
$x^{*}$	Current optimal estimate
$x_{\min}$	Lower limit of the variable (thermal conductivity), ${\mathrm{Wm}}^{-1}K^{-1}$
$x_{\max}$	Upper limit of the variable (thermal conductivity), ${\mathrm{Wm}}^{-1}K^{-1}$
$x_{\mathrm{mid}}$	Middle of the range of the variable (thermal conductivity), ${\mathrm{Wm}}^{-1}K^{-1}$
X	Training input set
y	Objective function values with noise
Y	Training output set
Greek symbols	
ε	Observation noise
$\zeta_{1}$	The accuracy of the range evaluation function
$\zeta_{2}$	The accuracy of the objective function
$\theta_{1}$, $\theta_{2}$	Two hyperparameters
Θ	Source item
κ	Thermal diffusivity, ${\mathrm{mm}}^{2}s^{-1}$
λ	Thermal conductivity, ${\mathrm{Wm}}^{-1}K^{-1}$
$\mu_{t}\left(x\right)$	The expectation of the Gaussian distribution at x
ξ	Equilibrium parameter
ρ	Density, ${\mathrm{kgm}}^{-3}$
σ	Variance
$\sigma_{t}\left(x\right)$	The variance of the Gaussian distribution at x
τ	Dimensionless time
$\phi \left(\cdot \right)$	The cumulative density function
$\Phi \left(\cdot \right)$	The standard Gaussian probability density function
χ	Observation space
Subscripts	
HS	A hypothetical solid specimen
Kap	Kapton layer
Ni	Nickel heater
S	Solid specimen
Abbreviations	
AMIP	Analytical model identification program
BOA	Bayesian optimization algorithm
BOAAIP	Bayesian optimization algorithm with an adaptive initial population
CFD	Computational fluid dynamics software
FENM	Finite element numerical model
GA	Genetic algorithm
MCMC	Markov Chain Monte-Carlo
TPS	Transient planar source
